# Cellular responses to modified *Plasmodium falciparum *MSP1_19 _antigens in individuals previously exposed to natural malaria infection

**DOI:** 10.1186/1475-2875-8-263

**Published:** 2009-11-23

**Authors:** Christian MF Okafor, Chiaka I Anumudu, Yusuf O Omosun, Chairat Uthaipibull, Idowu Ayede, Henrietta O Awobode, Alex B Odaibo, Jean Langhorne, Anthony A Holder, Roseangela I Nwuba, Marita Troye-Blomberg

**Affiliations:** 1 Cellular Parasitology Programme, Department of Zoology University of Ibadan, Ibadan, Nigeria; 2 Oni Memorial Children's Hospital, Ring Road, Ibadan, Nigeria; 3 Division of Parasitology, MRC National Institute for Medical Research, Mill Hill, London NW7 1AA, UK; 4 Department of Immunology, Wenner-Gren Institute, Arrhenius Laboratories, Stockholm University, Stockholm, Sweden; 5 College of Art and Sciences, Northwest University, 5520, 108th Ave. NE, Kirkland WA 98033, USA; 6 Department of Biotechnology, Bells University of Technology, Sango-Otta, Nigeria; 7 Protein-Ligand Engineering and Molecular Biology Laboratory, National Center for Genetic Engineering and Biotechnology (BIOTEC), Thailand Science Park, Pathumthani, Thailand

## Abstract

**Background:**

MSP1 processing-inhibitory antibodies bind to epitopes on the 19 kDa C-terminal region of the *Plasmodium falciparum *merozoite surface protein 1 (MSP1_19_), inhibiting erythrocyte invasion. Blocking antibodies also bind to this antigen but prevent inhibitory antibodies binding, allowing invasion to proceed. Recombinant MSP1_19 _had been modified previously to allow inhibitory but not blocking antibodies to continue to bind. Immunization with these modified proteins, therefore, has the potential to induce more effective protective antibodies. However, it was unclear whether the modification of MSP1_19 _would affect critical T-cell responses to epitopes in this antigen.

**Methods:**

The cellular responses to wild-type MSP1_19 _and a panel of modified MSP1_19 _antigens were measured using an *in-vitro *assay for two groups of individuals: the first were malaria-naïve and the second had been naturally exposed to *Plasmodium falciparum *infection. The cellular responses to the modified proteins were examined using cells from malaria-exposed infants and adults.

**Results:**

Interestingly, stimulation indices (SI) for responses induced by some of the modified proteins were at least two-fold higher than those elicited by the wild-type MSP1_19_. A protein with four amino acid substitutions (Glu27→Tyr, Leu31→Arg, Tyr34→Ser and Glu43→Leu) had the highest stimulation index (SI up to 360) and induced large responses in 64% of the samples that had significant cellular responses to the modified proteins.

**Conclusion:**

This study suggests that specific MSP1_19 _variants that have been engineered to improve their antigenicity for inhibitory antibodies, retain T-cell epitopes and the ability to induce cellular responses. These proteins are candidates for the development of MSP1-based malaria vaccines.

## Background

The development of an effective malaria vaccine remains a major public health challenge. Merozoite surface protein (MSP)-1 of *Plasmodium falciparum *is being developed as a vaccine candidate to protect against the erythrocytic stages of the malaria parasite [1,2]. Much of the work has been focused on the 19 kDa C-terminal region of MSP1 (called MSP1_19_[3]). Protection against challenge infection following immunization in rodent and monkey models of malaria has been reported [4-11]. However, sero-epidemiological studies [12-16] and vaccine trials [17] in human populations have given conflicting results concerning the protective role of anti-MSP1 antibodies, which may be explained by differences in the fine specificities of the MSP1_19_-specific antibodies [18,19]. It can be concluded from these studies that in the humoral control of malaria infection, the fine specificity of the antibody response may be crucial to inhibit erythrocyte invasion by merozoites.

The MSP1 precursor is cleaved into four fragments on the merozoite surface and at invasion the C-terminal 42 kDa fragment (MSP1_42_) is processed further into two smaller fragments: a 33 kDa polypeptide (MSP1_33_) and the C-terminal MSP1_19_, which remains on the parasite surface during invasion of red blood cells (RBC). MSP1 has been reported to elicit three types of antibody that can bind MSP1_42_[2,20,21]. These are a) inhibitory antibodies, which inhibit the cleavage of MSP1_42 _and thus invasion of RBC; b) blocking antibodies, which have overlapping specificities and compete with inhibitory antibodies for binding to the antigen, thereby allowing processing and invasion to occur even in the presence of inhibitory antibodies; and c) neutral antibodies that are neither inhibitory nor blocking. Significantly, it has been shown that all these types of MSP1_19_-specific antibodies are part of the natural immune response to MSP1 in malaria-exposed individuals [22,23]. Thus, the rational design of an MSP1-based malaria vaccine for the preferential induction of processing-inhibitory antibodies with the appropriate specificities is an important goal. The relative abundance of these protective antibodies in relation to the detrimental (blocking) antibodies in any infection is one of the important factors that may determine the outcome of that infection [2,3,20,22].

The MSP1_19 _epitopes recognized by inhibitory and blocking monoclonal antibodies (mAbs) have been mapped using site-directed mutagenesis, PEPSCAN, and nuclear magnetic resonance (NMR) [21,24,25]. A number of single and multiple amino acid substitutions in MSP1_19 _has been made, which had either no effect, or reduced, or completely abolished the binding of individual mAbs [21]. Recent data have shown that polyclonal antibodies in sera obtained from individuals living in a malaria endemic area recognize and bind to the modified antigens [22,23]. A vaccine based on one of these modified proteins could be designed to induce inhibitory but not blocking Abs and thus provide a focused polyclonal antibody response to inhibit RBC invasion and cleavage of MSP1 [21,22].

CD4^+ ^T-cell responses, providing help for MSP1-specific B-cell responses, are essential for protective immunity in rodent models of malaria, in protective immunity induced by immunization with MSP1_19_[26]. Since it is possible that the amino acid substitutions may alter the pattern and kinetics of MSP1_19 _antigen processing of within the MHC class II pathway, and thus the peptides presented, it will be important to determine whether the variant MSP1 molecules are recognized by immune cells obtained from individuals naturally primed by malaria infection. Some reports have suggested that T-cell responses to MSP1 in malaria-exposed donors are poor [27,28], possibly because of the difficulty in processing the highly disulfide bonded globular MSP1_19 _[27,29,30]. Also, the common occurrence of high background responses in both unexposed and exposed donors makes it difficult to demonstrate T-cell responses in field samples. The study described here was carried out to determine whether modified MSP1_19 _antigens were recognized by immune cells from naturally exposed and immune individuals living in a malaria endemic area, and whether modification of the critical T cell epitopes of MSP1_19 _would enhance or compromise cellular responses. Results from this study show that variant MSP1_19 _with two or more amino acid substitutions not only stimulated PBMC from exposed individuals but also induced proliferative responses *in vitro *of greater magnitude than the wild-type (WT) antigen. These results suggest that these modified MSP1 proteins may be suitable candidates for a malaria vaccine that would induce both protective antibodies and suitable cellular responses.

## Methods

### Subjects and samples

Volunteers were recruited in Ibadan, Nigeria where *P. falciparum *malaria is perennial. High malaria transmission occurs from April to November, corresponding to the rainy season (March to October), all samples were collected in March. The study protocol was reviewed and approved by the Joint University of Ibadan/University College Hospital Ethical committee. Informed consent was obtained from all participating individuals or legal guardians of children enrolled in the study. The donors were divided into two groups; the first was between three months and seven years of age (six individuals) and had clinical malaria symptoms, while the second aged between 13 yrs and 43 yrs (30 individuals) was without clinical malaria at recruitment. Venous blood (up to 4 ml from children and 7 ml from adults) was collected in heparinized tubes and transported immediately in insulated boxes to the laboratory for processing at the Department of Immunology, Wenner-Gren Institute, Stockholm University, Sweden. Giemsa-stained thick-blood films were examined for the presence of malaria parasites. A group of seven malaria-unexposed European donors provided control samples for the study.

### Peripheral blood mononuclear cells (PBMC)

Human PBMC were harvested from whole blood approximately 24 hrs after phlebotomy. PBMC were prepared by Ficoll-Hypaque (Pharmacia, Uppsala, Sweden) density gradient centrifugation according to the manufacturers' instructions. The harvested cells were suspended in freezing medium [10% dimethyl sulfoxide (DMSO), 20% fetal calf serum (FCS), RPMI 1640, HEPES and L-glutamine], frozen at 1°C min^-1 ^to -70°C in plastic containers (Nalgene Cryo, Nalge Company, USA), and then transferred into liquid nitrogen until used in *in vitro *assays. The cells had a demonstrated >95% viability when compared with frozen cells collected in Sweden.

### Antigens

Cells in the proliferation assays were stimulated by antigens at a predetermined final concentration of 1 μg/ml.

#### Wild-type (WT) and modified MSP1 proteins

The recombinant wild-type and modified MSP1_19 _antigens fused to *Schistosoma japonicum *glutathione S-transferase (GST) have been described previously [21]. Table 1 shows the specific modifications in the primary sequence of the MSP1_19 _antigens used; residues are numbered from the N-terminus of MSP1_19_.

**Table 1:** The panel of modified MSP1_19 _antigens

Amino acid substitutions in the primary sequence		
**Position(s)**	**Wildtype residue**	**Variant residue**

*Single amino acid*		

6	Gln	Ile

14	Gln	Arg

32	Leu	Arg

33	Asn	Ile

40	Lys	Ile

*Multiple amino acid*s		

12+28	Cys+Cys	Ile+Trp

34+39	Tyr+Asp	Ser+Thr

43+48	Glu+Thr	Leu+Lys

15+27+31+43	Asn+Glu+Leu+Glu	Arg+Tyr+Arg+Leu

27+31+34+43	Glu+Leu+Tyr+Glu	Tyr+Arg+Ser+Leu

Position in the sequence is numbered from the N-terminus of MSP1_19_

#### *P. falciparum *antigen

Crude *P. falciparum *antigen (F32) was used as a positive control for confirming previous infection or lack of exposure to *P. falciparum *in exposed and naïve individuals [31].

#### Control antigens

Phytohemagglutinin (PHA; Sigma, Poole, UK) was used as positive control for testing lymphocyte viability and responsiveness. Recombinant GST was used as a control antigen.

### Cell proliferation assays

Frozen PBMCs were thawed, washed three times, and resuspended at a final concentration of 10^6 ^viable cells/ml in complete medium [RPMI 1640 containing 2 mM L-glutamine, 50 U/ml penicillin, 0.1 mg/ml streptomycin, 10 mM HEPES, 0.22% (vol/vol) sodium bicarbonate and 10% heat-inactivated non-immune FCS]. The cells were then plated into sterile, round-bottomed 96-well microtiter plates at 10^5 ^viable cells/well in 100 μl culture medium. Antigens were diluted to 2 μg/ml in complete medium and 100 μl of the antigen solution added to the wells containing the cells (in triplicates), giving a final antigen concentration of 1 μg/ml. Unstimulated cultures served as negative controls, and those stimulated with PHA as positive controls. The plates were incubated at 37°C in a humidified atmosphere containing 5% CO_2 _for 6 days. On the 5^th ^day of incubation, 100 μl of supernatant were removed from each well and replaced with 100 μl of fresh medium containing [^3^H] thymidine (1 μCi/well; Amersham Life Sciences, Little Chalfont, UK) for a further 16-18 hours. Cells were harvested onto filter mats using a cell harvester (Tomtec) and incorporation of radioactivity was measured using a Microbeta counter. Proliferative responses expressed as the geometric mean of radioactivity incorporation in counts per minute were determined for each antigen, and the stimulation index (SI) was calculated as the ratio of incorporation by antigen-stimulated cells to that of the unstimulated control cells. Subjects whose cells responded to one or more variants with a SI two-fold or more above that of the GST control were grouped as responders whereas others with a SI similar to or lower than that of GST were not considered to be responders. This cut-off was set to ensure that we were measuring a response to MSP1_19 _and not GST.

### Statistics

Correlation coefficients and stimulation indices were plotted using SPSS and MS Excel software (*p *= 0.05 was considered significant).

## Results

### Parasitemia of donors

Microscopic examination of blood smears from the 36 donors in this study showed that 20 (56%) were positive for *P. falciparum*, while no parasites were detected in the blood of the remaining donors. Parasite burden in individuals decreased significantly with increasing age among the subjects (*p *= 0.021). Among the parasitemic subjects, 6 of 20 were symptomatic at the time of recruitment with parasite densities ranging between 280 and 72,727 parasites/μl blood, while the remaining 14 donors who were asymptomatic had a parasitemia that ranged between 40 and 600 parasites/μl blood (data not shown).

### Cellular proliferation in response to the wild-type and modified MSP1_19 _antigens

**Figure 1:**
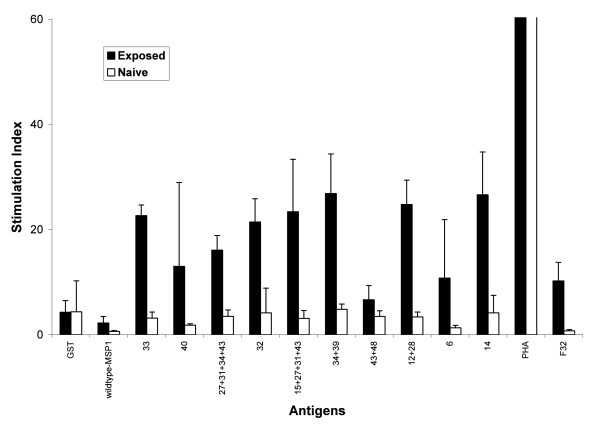
**Mean T cell responses in malaria-exposed and un-exposed individuals**. Responses to the wildtype-MSP1_19_, modified MSP1_19 _antigens, PHA, GST and F32 (a crude malarial antigen preparation) in seven malaria-exposed individuals (shaded boxes) and five unexposed malaria-naïve individuals (open boxes), as measured by stimulation index (SI). The malaria-exposed individuals showed significantly higher responses to all the malaria antigens than the malaria-unexposed individuals (*P *= 0.001).

The stimulatory capacity of each antigen in the panel was assessed. *In vitro *cellular responses to the WT MSP1_19 _and a panel of ten variants with one or more amino acid substitution [21] (Table 1) were studied, using PBMC from the 36 naturally exposed and 7 malaria-naïve donors. Proliferative responses to both the WT and the modified antigens were observed and a stimulation index was calculated for each. Though not all the malaria-exposed donors responded to both WT and modified antigens, these individuals had significantly higher proliferative responses than the malaria naïve group (Figure 1). In addition, for the malaria-exposed individuals, it was observed that most of the responses to the modified MSP1_19 _antigens were significantly greater than the response to the WT molecule.

**Figure 2:**
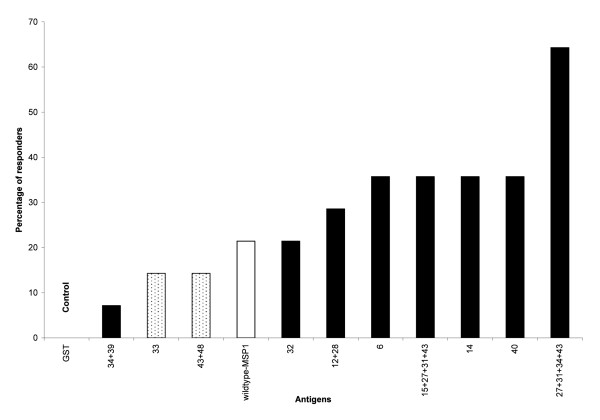
**Prevalence of response to MSP1_19 _variant and wildtype antigens**. Prevalence of T cell responses to the antigens for 14 individuals that were identified as high responders. The observed responses to the variants (shaded bars) were significantly higher than the response to the WT-MSP1_19 _(open bar). The responses to the variants 43+48 and 33 were higher than that to the GST-control but lower than that to WT-MSP1_19_.

Cells from 14 (39%) of the exposed individuals had a significant proliferative response to the panel of MSP1_19 _antigens (SI range >2 - 360). These individual were grouped as responders, while the remaining 61% of malaria-exposed donors that responded poorly to the antigens were grouped as non-responders. A modified protein, 27+31+34+43 with four amino acid substitutions (Table 1), had the most intense and consistently highest T-cell stimulatory effect, with a mean SI as high as 360 and elicited a response in the greatest number of donors: nine of 14 (64%) responders had a significant response to this variant (Figure 2). In these same nine individuals, the responses to the 27+31+34+43 variant were significantly higher than the responses to the wild-type (WT-MSP1_19_) antigen. Using cells collected from the same individual the SI was 240-fold greater than the SI seen in response to WT-MSP1. More than half of the nine (5/9) individuals that responded to the 27+31+34+43 variant had a SI that was at least five-fold higher than that seen in response to the wild-type antigen.

Three single amino acid substitution variants 6, 14 and 40 (Table 1) and the 15+27+31+43 variant (a four-residue substitution variant) all elicited high responses in 5/14 (36%) of the responders (Figure 2). Four of 14 (27%) responders had a significant response to the 12+28 variant which lacks a disulfide bond (Cys12→Ile and Cys28→Trp). The 34+39 variant (Tyr34→Ser, Asp39→Thr) elicited a response in only one of the responders. Two of the 14 (14%) responders had responses to the 33 (Asn33→Ile) and 43+48 (Glu43→Leu and Thr48→Lys) variants; the intensity of these responses were higher than the responses to the GST control protein but lower than the responses to WT-MSP1_19_.

Intense responses were also elicited by the 15+27+31+43 variant in five of the responders with a mean SI of 314.6. Three out of six individuals that responded to 15+27+31+43 had a SI that was at least five-fold greater than that to the wild-type protein. Variants 14 and 12+28 induced a mean SI of 315.7 and 269, respectively. The data revealed that all the high stimulation indices (SI >100), occurred with the MSP1_19 _variants containing two or more amino acid substitutions except for the variant 14 which had a single amino acid change of Gln to Arg at position 14. All the variants in the panel that induced an intense response consistently induced a ≥3-fold stronger response than the WT protein.

**Figure 3:**
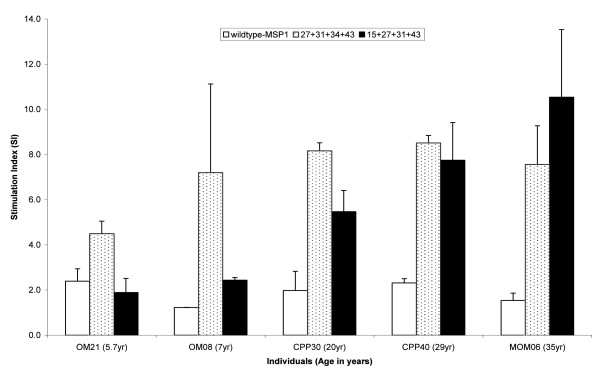
**Cellular responses to wildtype-MSP1_19 _antigen and the two variants with four amino acid substitutions**. 27+31+34+43 and 15+27+31+43 MSP1_19 _variants in 5 individuals of different ages and malaria exposure. OM21 and OM08 had clinical presentation but the others did not. The stimulation indices (SI) for the 27+31+34+43 (dotted boxes) and 15+27+31+43 (shaded box) variants were significantly higher than the SI for the WT-MSP1_19 _antigen (clear boxes).

The difference between the two 4-amino acid substitution variants, 27+31+34+43 and 15+27+31+43 is a substitution Asn15→Arg in 15+27+31+43 and Tyr34→Ser in 27+31+34+43. The responses to these two variants were compared in five individuals that responded to both variants (Figure 3). For this small group, the data suggested that the 27+31+34+43 variant preferentially induced a response in cells obtained from children whereas 15+27+31+43 had a greater effect with cells from adults. The response to the WT antigen did not change significantly with increasing age (Figure 3).

## Discussion

This study investigated the proliferative cellular responses to wild-type MSP1_19 _and several variant proteins in individuals naturally exposed to the *P. falciparum *parasite. The results revealed that the modified MSP1_19 _antigens induced stronger and more frequent responses as compared to the wild type MSP1. In particular, the responses seen to most of the modified antigens with two or more amino acid substitutions were stronger than the response to the native antigen. These findings indicate that the antigenic epitopes in some of the MSP1_19 _variants were not compromised by the specific amino acid modifications; some of these modifications actually enhanced the response to the proteins.

The MSP-1_19 _sequence is highly conserved, largely with diversity at only four positions in its 96 amino acid residues [32]. This study has shown that selective replacement of up to four amino acid residues in the MSP1_19 _sequence did not compromise the linear epitopes that are recognized by T-cells.

In the population tested, a response to one or more variants was noted in 38% of individuals, whereas only 22% had a proliferative response to WT-MSP1_19_. In previous work to evaluate human T-cell responses to WT-MSP1_19 _antigen a prevalence of 26% responders was observed in Gambian and Kenyan populations, with marked individual variations [27,28].

The higher prevalence of cellular proliferative responses to the modified MSP1_19 _noted in this study might be explained by structural changes induced by the modifications. It has been suggested that the relatively poor cellular response to MSP1_19 _is due to its structural complexity and resistance to proteases. Thus with 6 disulfide bonds it is impossible to load MSP1_19 _onto MHC class II molecules and it can only be available for loading when the bonds have been reduced [29,30]. A form of MSP1_19 _that is more accessible to proteases would allow improved processing by antigen-presenting cells (APC), which could enhance the cellular responses and thus vaccine efficacy [29].

Human PBMC recognized and responded significantly to eight out of ten variants, which had been designed to either reduce or abolish the binding of known blocking mAbs [21] (Figure 1). Among those individuals whose cells responded, the prevalence of the responses to each variant ranged from 7% to 64% of the population. More people responded to the 27+31+34+43 variant than any of the other variants tested (Figure 2). The ability of the cells to respond to more than one variant was age dependent, thus cells from adults responded to more variants than the cells from children. This suggests a possible role of previous and repeated exposure to infection in the breadth of recognition and response to these variants (Figure 3).

The two mutant proteins with four amino acid substitutions designated 27+31+34+43 and 15+27+31+43, characterized as having epitopes for MSP1-processing inhibitory antibodies, but no binding affinity for known blocking mAbs [21] were the most frequently recognized and they induced some of strongest cell responses observed. The 27+31+34+43 mutant induced the strongest proliferative response (SI up to 360) and was the antigen most frequently recognized by cells from malaria-exposed adults and children. Each individual that responded to the 15+27+31+43 variant also responded either with equal or greater intensity to the 27+31+34+43 variant, suggesting a better recognition of the 27+31+34+43 variant by the responding cells. This is attributable to the Tyr34→Ser modification in the 27+31+34+43 MSP1_19 _variant compared with the Asn15→Arg substitution in the 15+27+31+43 variant. This result is consistent with the suggested role of Tyr34 at the interface between the two epidermal-growth factor (EGF) domains in providing structural stability; the replacement of tyrosine by serine may result in an antigen that can be processed more readily. From these results, and the earlier antigenicity studies, the 27+31+34+43 MSP1_19 _variant would be a favored variant for development of a MSP1-based vaccine. However, additional studies are needed to determine whether the difference in the cellular responses to these two closely related proteins is a result of recognition by individual cell types (host factors) or a result of the structural perturbations due to the amino acid substitutions in the MSP1 sequence.

## Conclusion

This study provides a possible basis to support the use of the 27+31+34+43 and 15+27+31+43 MSP1_19 _proteins as engineered immunogens to induce improved cellular responses and provide the T-cell help for the production of protective MSP1 processing-inhibitory antibodies. Unlike the wild-type MSP1_19 _protein it is expected that these proteins will not induce blocking antibody specificities. However, there is a need to carry out more detailed studies of cellular responses to MSP1_19 _variants with a larger sample size. In order to understand the importance of these modifications in antigen presentation, it would be useful to properly characterize the phenotypes of the proliferating cells. In addition the role of individual host genetics, and other factors in the modulation of cellular responses to these variants cannot be overemphasized. The potential *in vitro *correlates of protective immunity defined in this study represent an important step forward in the search for and evaluation of an efficient MSP1-based malaria vaccine.

## Abbreviations

GST: glutathione S-transferase; MSP: merozoite surface protein; PBMC: peripheral blood mononuclear cells; RBC: red blood cell; SI: stimulation index; WT: wildtype.

## Competing interests

The authors declare that they have no competing interests.

## Authors' contributions

CMFO, CIA, RIN, MTB AAH, CU, IA and JL made substantial contributions to the conception and the design of the study, or acquisition, analysis and interpretation of data; YOO, HOA and ABO were involved in drafting the manuscript or revising it critically for content; and RIN, JL, AAH and MTB, gave final approval of the version to be published.
